# Degree Correlations Optimize Neuronal Network Sensitivity to Sub-Threshold Stimuli

**DOI:** 10.1371/journal.pone.0121794

**Published:** 2015-06-26

**Authors:** Christian Schmeltzer, Alexandre Hiroaki Kihara, Igor Michailovitsch Sokolov, Sten Rüdiger

**Affiliations:** 1 Institut für Physik, Humboldt-Universität zu Berlin, Germany; 2 Universidade Federal do ABC, Santo André, Brazil; University of Zaragoza, SPAIN

## Abstract

Information processing in the brain crucially depends on the topology of the neuronal connections. We investigate how the topology influences the response of a population of leaky integrate-and-fire neurons to a stimulus. We devise a method to calculate firing rates from a self-consistent system of equations taking into account the degree distribution and degree correlations in the network. We show that assortative degree correlations strongly improve the sensitivity for weak stimuli and propose that such networks possess an advantage in signal processing. We moreover find that there exists an optimum in assortativity at an intermediate level leading to a maximum in input/output mutual information.

## Introduction

Revealing the neural code is one of the most ambitious goals in neuroscience. The theory of complex networks is a promising approach to this aim: high cognitive processes are treated as emergent properties of a complex connectivity of many ‘simple’ neurons. An important question is then how the network structure relates to the collective activity of the connected neurons and how this activity can be interpreted in terms of neuronal coding and processing strategies. Advanced techniques for visualization of the activity of single neurons and groups of neurons allow for identification of synaptic links between neurons, leading to the possibility of inferring statistical network characteristics. Neuronal network topology is far from being completely random [1, 2]. Among the most intriguing topological properties are small-world features [3], modularity [4] and large variations in connectivity, e.g. scale-free functional structure [5] and the presence of strongly connected and highly active hubs [6–8]. How are these network properties related to neuronal dynamics? Although the influence of network parameters on the dynamics of neuronal populations has been subject to a large number of studies [1, 9–15], the relevance of heterogeneous connectivity and higher order network statistics have been investigated only recently [12–16]. Simulations show that heterogeneous connectivity structures induce heterogeneous activity patterns [12–16]. Indeed, activity in the neocortex is highly variable: A large proportion of neurons fires at very low rates, whereas a small subset of neurons is highly active and better connected [17–20]. Variability in neuronal network structure and dynamics may be important for information processing such as the detection of weak input signals [12, 15, 20, 21]. Remarkably, some brain regions, such as the barrel cortex, can sense very weak stimuli down to a few spikes to a single neuron [22]. It has been hypothesized, that a sub-set of highly active and highly interconnected neurons may play a significant role in the encoding of sensory information in this region [20].

Here, we study the relevance of two basic network topological features, the degree distribution and degree correlations, for the ability of a network to sense and amplify small input signals. A first approach requires a simple mathematical model describing the activity of neurons. As a basic yet very relevant representation of neuronal dynamics, we here consider rate coding in a network of leaky integrate-and-fire (LIF) neurons and use a mean-field model to account for their synaptic coupling. The powerful mean-field theory rests on the assumption that neurons interact through their synaptic activity but generally fire asynchronously. The information contained in the neuronal network is encoded in their mean firing rate [23, 24]. At this level, the ability of the network to process information is shaped by the relationship of the input signal in form of an input spike frequency and the resulting firing rate of the network. The former is an independent Poissonian spike train injected synaptically into all neurons and the latter can be characterized as an output signal by averaging over the entire population or a part of the population.

This mapping of inputs to the average activity of many neurons has been described as one of the fundamental processing strategies of the brain [25]. One of the major advantages of this *population coding* is its robustness to failure as information is encoded across many cells. Population coding is important in many brain functions such as orientation discrimination in the primary visual cortex [26], control of eye [27] and arm movements [28]. How the brain can access and process information encoded in the collective activity and how it manipulates this activity to perform these computations efficiently is one of the main questions in computational neuroscience. We aim at this problem by calculating the input/output relationship of large LIF networks using a generalized mean-field method, which takes into account the complex network topology. Furthermore, we are interested in the ability of the network to convey information about stimuli of small amplitude. For weak stimuli, where afferent injected currents are too small to trigger significant single neuron firing, collective activity of the network strongly depends on recurrent input [16, 29], and it may be expected that the effect of recurrent network structure on signal processing is most pronounced in this regime.

**Fig 1:**
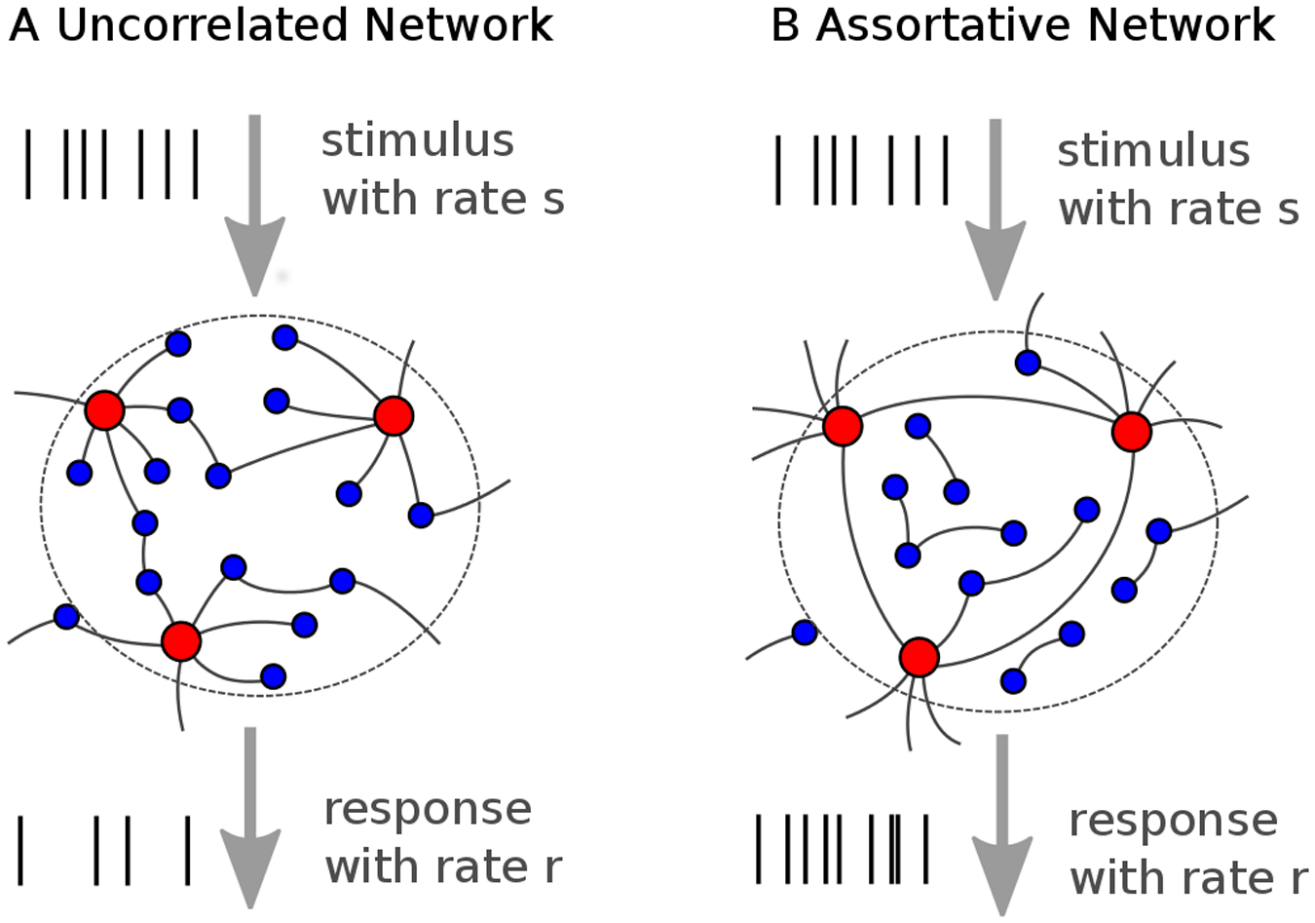
Schematic of the heterogeneous neuronal networks. (A) In the uncorrelated network, highly connected neurons and poorly connected neurons are joined randomly. Here, red nodes represent an exemplary well connected subpopulation, while blue nodes represent all remaining populations with smaller in-degree *k*. (B) In the assortative network, neurons with similar connectivity are joined preferably. The network is stimulated by Poissonian external input spike trains with mean rate *s*, which are injected to each neuron. The network response *r* to the stimulus is quantified by the average firing rate of a randomly chosen fraction of the network (*n* neurons).

To describe collective neuronal activity one often uses the population-density approach, which was successfully applied to cortical circuits of identical neurons [9, 30–32] and to networks of heterogeneous neurons [21]. In the population density approach, the spiking and interplay of many neurons, for instance in the network of a single cortical column, is captured by a probability density function for the states of statistically similar neurons [24, 31]. Here we extend this theory to include the heterogeneity of the network in terms of the degree of a given neuron, i.e., the number of synaptic connections the neuron possesses. We find that the network’s heterogeneity leads to substantial deviations from simple mean-field calculations, where one ignores the network properties. Our method is to divide the whole neuronal population in subpopulations according to the number of incoming synaptic links, or the *in-degree* *k*, of neurons. This allows us to consider networks with different levels of assortativity with respect to *k* (Fig 1), which is a measure of the correlations in the degree of nodes [33]. Degree correlations in neural networks may result from a number of processes including plasticity and they are interesting for a number of reasons. First, degree correlations can be considered the most basic statistical property of a complex network except for the degree distribution itself. Second, there have been large efforts devoted to understand correlations in neuronal spiking [34], but the effects of correlations in structural connectivity have been much less studied so far. Finally, statistical network properties have been considered in the context of synchronization [35], but the characterization of spiking in the unsynchronized regime has not received similar attention in the context of complex networks.

Using the subpopulation density approach, we calculate the firing rates for the coupled *k-populations* and show that the obtained values exhibit excellent agreement with results of direct numerical simulation of a network of LIF-neurons. In-degree distribution and degree correlations of the network enter the equations in form of a joint degree distribution *N*_*kk*′_, which is the average number of connections from a *k*′- to a *k*-neuron. To be specific in this analysis, we derive simple expressions for *N*_*kk*′_ for exemplary networks of various degree correlation types in the limit of large network size. We apply our method to a correlated network with broad degree distribution and show that the shape of the input/output relationship is strongly affected by different levels of assortativity. Assortative networks sustain activity at sub-threshold levels of input, where uncorrelated networks would not fire, and thus, are more sensitive to weak stimuli. Hence, assortativity may tune the input/output relationship to the distribution of inputs and noise of the system to optimize information transfer. Finally, we calculate the mutual information between sub-threshold stimuli and network response, assuming that each stimulus is equally likely. Recently, mutual information has been used in a similar way to investigate information transmission in cortical networks with balanced excitation and inhibition [36]. For our model network, an excess of assortativity leads to an increase in noise in the firing rate and an optimum of input/output mutual information is found in a range of assortativity consistent with recent estimates from neuronal cultures [37, 38]. Preliminary results of our investigations were published in abstract form in [39].

## Results

### Meanfield Method for Heterogeneous Correlated Networks

#### Deriving the Self-Consistent Equations

We begin with a mean-field method for calculating the stationary firing rate of a heterogeneous LIF network with degree correlations. Assume a network of LIF-neurons with heterogeneously distributed in-degrees (Fig 1). Each neuron is stimulated with an independent external Poisson spike train with rate *s* quantifying the input signal or stimulus strength. If the input strength is small, an unconnected neuron will not reach the threshold for excitation, while for sufficiently large *s* the neuron will fire. Following Brunel [9], we normalize *s* by the rate *ν*_thr_ (see below) that is needed for a neuron to reach threshold in the absence of feedback. For *s* ≥ 1 the network is mostly driven by the external input, whereas for *s* < 1 the response is dominated by sustained recurrent activity. The network response *r* is quantified by the average firing rate of a subset *n* of its neurons and can be considered a feed-forward activity of the neural population or output.

We are interested in the dependence of the input/output relationship on degree correlations in the network. Since the network of neuronal connections is directed, we have to distinguish between the number of incoming and outgoing connections of a neuron, its *in-degree* *k* and *out-degree* *j*. In the following we mainly focus on networks with *k* = *j*, suggested by findings for *C. elegans* [40], where in- and out-degree per neuron strongly correlated. The distribution of in- and out-degrees of the neurons is denoted by *P* ^in^(*k*) and *P* ^out^(*j*), respectively. Furthermore, we take into account in-degree correlations. Main attention is paid to the assortative case when neurons with high in-degree preferably connect to other neurons with high in-degree. Such correlations have been found in cortical networks and in neuronal cultures [5, 37, 41].

The LIF dynamics of a neuron in the population is governed by a single state quantity. The depolarization *V*_*i*_(*t*) of neuron *i* follows the ordinary differential equation

$$\tau \frac{dV_{i}\left(t\right)}{dt}=-V_{i}\left(t\right)+R\left(I_{i}^{\text{rec}}+I_{i}^{\text{ext}}\right),$$ (1)

where *τ* is the membrane time constant, *R* is the membrane resistance and $I_{i}^{\text{rec}}$ and $I_{i}^{\text{ext}}$ are the recurrent and external input, respectively. If the depolarization *V*_*i*_(*t*) reaches a threshold value Θ, the neuron fires an action potential and *V*_*i*_(*t*) is reset to a refractory voltage *V*_*r*_ for a constant refractory period *τ*_ref_. Since the neuron cannot fire in this time interval, its maximum firing rate is 1/*τ*_ref_. Action potentials arriving at the *i*-th neuron are modeled as delta spikes contributing to the recurrent synaptic current

$$RI_{i}^{\text{rec}}\left(t\right)=\tau J\sum_{j=1}^{N}a_{ij}\;\sum_{l}\;\delta \left(t-t_{j}^{l}-D_{ij}\right)$$ (2)

where *J* is the postsynaptic amplitude, and *a*_*ij*_ is the adjacency matrix of the network. The second sum runs over all pulses *l* fired by neuron *j* at times $t_{j}^{l}$. These arrive at neuron *i* after a short synaptic delay *D*_*ij*_.

Let us consider the dynamics of neurons in a *k*-population assuming that they receive uncorrelated inputs. In the limit of a large network the Poisson pulse flow can be replaced by a Gaussian process by means of the central limit theorem. Then, the input current of the *i*-th neuron is given by

$$RI_{i}^{\left(k\right)}\left(t\right)=\mu_{k}\left(t\right)+\sigma_{k}\sqrt{\tau }\xi_{i}^{\left(k\right)}\left(t\right),$$ (3)

with a mean *μ*_*k*_(*t*) and a fluctuating part (noise) with intensity *σ*_*k*_. Here *ξ*(*t*) is Gaussian white noise with zero mean and unit variance. Each *k*-population receives inputs from other *k*′-populations: *μ*_*k*_(*t*) and *σ*_*k*_ depend on the firing rates *r*_*k*′_ of other populations. Using Eq (3) we rewrite Eq (1) as a Langevin equation for $V_{i}^{\left(k\right)}(t)$:

$$\tau \frac{dV_{i}^{\left(k\right)}\left(t\right)}{dt}=-V_{i}^{\left(k\right)}\left(t\right)+\mu_{k}\left(t\right)+\sigma_{k}\sqrt{\tau }\xi_{i}^{\left(k\right)}\left(t\right).$$ (4)

The voltage distribution *P*(*V* ^(*k*)^) is given by the corresponding Fokker-Planck equation, and the average stationary firing rate ${\hat{r}}_{k}$ (given by the self-consistency conditions, see materials and methods) is given by

$$\begin{array}{cc} {\hat{r}}_{k} & ={\left[\tau_{\text{ref}}+\tau \sqrt{\pi }\int_{\left(V_{\text{reset}}-\mu_{k}\right)/\sigma_{k}}^{\left(\Theta -\mu_{k}\right)/\sigma_{k}}e^{x^{2}}\left(1+\text{erf}\left(x\right)\right)dx\right]}^{-1}\\ & =\phi_{k}\left({\hat{r}}_{k_{\text{min}}},\cdots ,{\hat{r}}_{k_{\text{max}}},s\right),\;k_{\text{min}}\leq k\leq k_{\text{max}}, \end{array}$$ (5)

where $\text{erf}(x)=\frac{2}{\sqrt{\pi }}\int_{0}^{x}e^{-y^{2}}\text{d}x$ is the error function, *k*_min_, *k*_max_ are the minimum and maximum degree in the network, and $\phi_{k}({\hat{r}}_{k_{\text{min}}},\ldots ,{\hat{r}}_{k_{\text{max}}},s)$ is the coupled transfer function. Thus, the steady-state firing rate of each *k*-population is coupled to the firing rates of all other *k*′-populations via the input mean and variance

$$\mu_{k}\left(t\right)=J\tau \;(\nu_{\text{thr}}s+\sum_{k^{\prime }}\;N_{kk^{\prime }}{\hat{r}}_{k^{\prime }}\left(t\right)),$$ (6)

$$\sigma_{k}^{2}\left(t\right)=J\cdot \mu_{k}\left(t\right),$$ (7)

where *N*_*kk*′_ is the average number of *k*′-neurons that synapse into a *k*-neuron (a joint degree distribution) and $\nu_{\text{thr}}=\frac{\Theta }{J\tau }$ [9]. Given *N*_*kk*′_, all ${\hat{r}}_{k}$ result by numerically solving Eq (5). *N*_*kk*′_ follows from the adjacency matrix of the network by averaging the number of links from *k*′-neurons to a *k*-neuron. In general, it is of the form

$$N_{kk^{\prime }}=kf\left(k,k^{\prime }\right),$$ (8)

where *f*(*k*, *k*′) is the probability that an incoming link of a *k*-neuron originates from a *k*′-neuron. Normalization requires

$$\sum_{k^{\prime }}\;f\left(k,k^{\prime }\right)=1,\;\sum_{k^{\prime }}\;N_{kk^{\prime }}=k.$$ (9)

#### Solving the Self-Consistent Equations

The approximate dynamics of the neuronal network can be described by the differential equation

$$\tau_{x}\frac{d}{dt}\hat{r}=-\hat{r}+\Phi \left(\hat{r},s\right),$$ (10)

where $\hat{r}$ and **Φ** are the vectors of population firing rates ${\hat{r}}_{k}$ and coupled transfer functions $\phi_{k}\left(\hat{r},s\right)$, and *τ*_*x*_ is a time-constant of appropriate choice [24, 31]. Solutions of the self-consistent Eq (5) are fixed points of the above equation and can be found by numerical integration. In order to integrate Eq (10), one needs to evaluate the input mean and variance of Eqs (6 and 7), where the joint degree distribution *N*_*kk*′_ enters. Thus, it is necessary to first assess the network topology by means of inferring this joint degree distribution. In the following, we will derive particularly simple expressions of *N*_*kk*′_ = *kf*(*k*, *k*′) in the thermodynamic limit for networks with arbitrary degree distributions and different in-degree correlations.

First assume a maximally random network of *N* neurons, where in- and out-degree of each neuron are drawn independently from the in- and out-degree distributions *P* ^in^(*k*) and *P* ^out^(*j*), respectively. In this case, in-degree correlations are minimal and *f*(*k*, *k*′) is simply the fraction $\frac{E_{k^{\prime }}}{E}$ of links that originate from neurons with in-degree *k*′, where *E* = *N*⟨*k*⟩ = *N*⟨*j*⟩ is the total number of links in the network. The size of a *k*′-population corresponds to the total number of *k*′-neurons in the network, which is *NP* ^in^(*k*′). A fraction *P* ^out^(*j*′) of this population has *j*′ outgoing links. Hence, the number of outgoing links of this fraction is

$$j^{\prime }P^{\text{out}}\left(j^{\prime }\right)N\;P^{\text{in}}\left(k^{\prime }\right).$$ (11)

We now sum up the outgoing links of all fractions of the *k*′-population to obtain the total number of outgoing links from all *k*′-neurons

$$E_{k^{\prime }}=N\;P^{\text{in}}\left(k^{\prime }\right)\sum_{j^{\prime }}j^{\prime }P^{\text{out}}\left(j^{\prime }\right)=N\cdot P^{\text{in}}\left(k^{\prime }\right)\left\langle j^{\prime }\right\rangle .$$ (12)

Thus, the fraction of links $\frac{E_{k^{\prime }}}{E}$ that originate from neurons with in-degree *k*′ is

$$f\left(k,k^{\prime }\right)=P^{\text{in}}\left(k^{\prime }\right).$$ (13)

The input mean and variance of Eqs (6 and 7) become

$$\mu_{k}\left(t\right)=J\tau \;(\nu_{\text{thr}}s+k\hat{r}),$$ (14)

$$\sigma_{k}^{2}\left(t\right)=J\cdot \mu_{k}\left(t\right),$$ (15)

where $\hat{r}$ is the mean firing rate of the network

$$\hat{r}=\sum_{k}\;P^{\text{in}}\left(k\right){\hat{r}}_{k}.$$ (16)

Thus, the self-consistent Eq (5) for the mean-firing rate of the network decouple and reduce to

$${\hat{r}}_{k}=\phi_{k}\left(\hat{r}\right),$$ (17)

and we can write a one-dimensional self-consistent equation for the mean firing rate $\hat{r}$ of the network

$$\hat{r}=\sum_{k}\;P^{\text{in}}\left(k\right)\phi_{k}\left(\hat{r}\right).$$ (18)

Interestingly, Eq (18) does not depend on the out-degree distribution of the network, because effects of its heterogeneity are averaged out in the limit of large network size.

In the following we assume random networks with in-degree distribution *P* ^in^(*k*) and equal in- and out-degree per neuron, *j* = *k*. Synaptic connections show no in-degree correlations if they are drawn at random, but now in-degree and out-degree of each neuron are positively correlated, as shown for the neuronal network of *C. elegans* [40]. In this case the number of outgoing links from all *k*′-neurons in the network is simply *k*′ times the number of *k*′-neurons

$$E_{k^{\prime }}=k^{\prime }N\;P^{\text{in}}\left(k^{\prime }\right).$$ (19)

The fraction of links $\frac{E_{k^{\prime }}}{E}$ that originate from neurons with in-degree *k*′ is then

$$f\left(k,k^{\prime }\right)=\frac{k^{\prime }}{\left\langle k\right\rangle }P^{\text{in}}\left(k^{\prime }\right).$$ (20)

This gives for the input mean and variance of Eqs (6 and 7), respectively

$$\mu_{k}\left(t\right)=J\tau \;(\nu_{\text{thr}}s+\frac{k}{\left\langle k\right\rangle }\sum_{k^{\prime }}\;k^{\prime }P^{\text{in}}\left(k^{\prime }\right){\hat{r}}_{k^{\prime }}\left(t\right)),$$ (21)

$$\sigma_{k}^{2}\left(t\right)=J\cdot \mu_{k}\left(t\right).$$ (22)

Thus, in a random network with equal in- and out-degree per neuron and without in-degree correlations of the synaptic connections, the input mean and variance of each *k*-population are dependent on the steady-state firing rate of all the other *k*′-populations. Note, that in networks without in-degree correlations, the probability *f*(*k*, *k*′) is always independent of *k*.

For networks with in-degree correlations, the average number of connections between a *k*′-population and a *k*-population differs from the uncorrelated case. In networks with assortative in-degree correlations, neurons preferably connect to neurons with similar in-degree. In the extreme case, the network segregates into disconnected subnetworks of neurons with the same in-degree:

$$f\left(k,k^{\prime }\right)=\delta \left(k,k^{\prime }\right),$$ (23)

where *δ*(*k*, *k*′) is the Kronecker delta. Note, that in networks with equal in- and out-degree per neuron the number of outgoing links and incoming links is equal for each *k*-population. Thus, in strongly assortative networks, all *k*-population subnetworks can be disconnected from each other. In networks with nonequal in- and out-degree per neuron, complete segregation into decoupled *k*-populations is only possible if the average number of outputs of *k*-neurons is *k*. A mismatch between the average number of inputs and outputs in a *k*-population results in connections between different populations and in this case, the distribution *f*(*k*, *k*′) may depend on the in- and out-degree distribution and, potentially, on correlations between in- and out-degree per neuron.

In networks with disassortative in-degree correlations, high-degree populations are connected to populations with low degree and vice versa. The dependence of the joint degree distribution on the probability distribution of the input and out-degrees can be calculated analytically for undirected networks [42]. In contrast to the assortative network, *f*(*k*, *k*′) here depends on the in-degree distribution of the network. Therefore, we sample *N*_*kk*′_ from the adjacency matrix of the disassortative network and show that our mean-field model accurately predicts its mean firing rate.

### Stimulus Response of Heterogeneous Correlated Networks

In what follows we use the *k*-population model to analyze the response of three representative heterogeneous networks with different degree correlations to a stimulus *s*. Theoretical predictions are then compared to full simulations of 10^5^ LIF neurons. For details on the model and theory, see materials and methods. We chose a power-law in-degree distribution of *P*(*k*) = *Zk* ^−2^ with normalization constant *Z* between a minimum degree *k*_min_ = 10 and a maximum degree *k*_max_ = 500, because we are interested in networks of strong heterogeneity. Power-law degree distributions have been found in recent *in vitro* experiments [37, 43, 44]. In-degree correlations are quantified by the Pearson correlation coefficient *p*, as defined in [45]

$$p=\frac{1}{\sigma_{\text{in}}^{2}}\;[\sum_{kk^{\prime }}kk^{\prime }e_{kk^{\prime }}-Q_{k}^{\text{in}}Q_{k^{\prime }}^{\text{in}}],$$ (24)

where *e*_*kk*′_ is the probability, that a randomly chosen directed link leads into a neuron of in-degree *k* and out of a neuron of in-degree *k*′. $Q_{k}^{\text{in}}$ is the excess in-degree distribution of the neuron at the origin of a random link

$$Q_{k}^{\text{in}}=\frac{\left(k+1\right)P^{\text{in}}\left(k+1\right)}{\sum_{k}kP^{\text{in}}\left(k\right)}.$$ (25)

**Fig 2:**
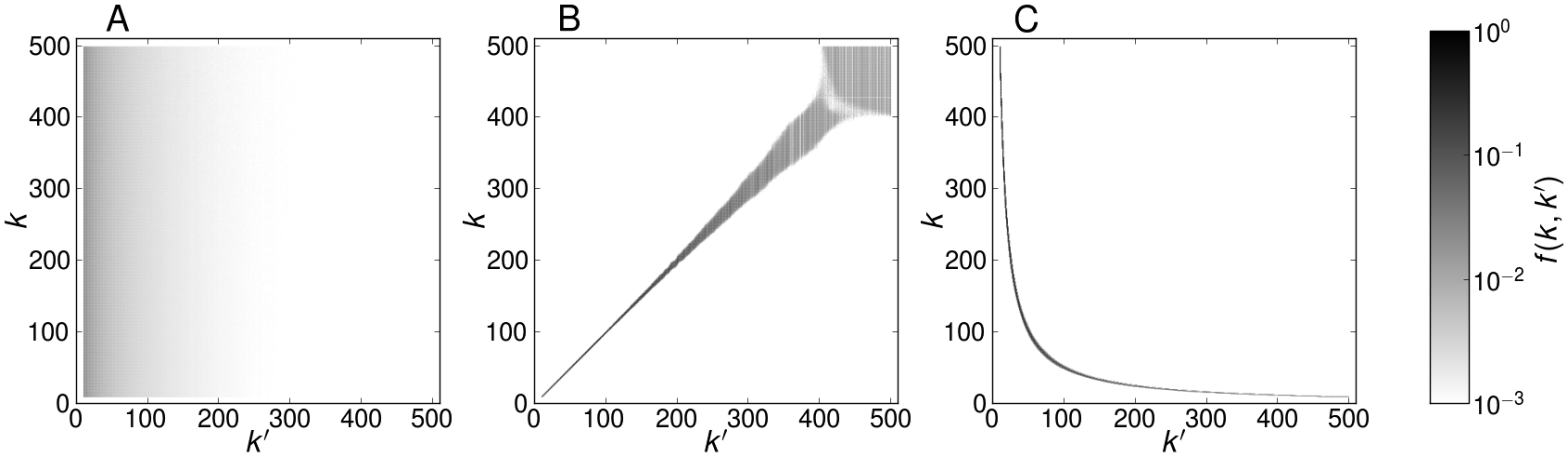
Probability *f*(*k*, *k*′) for a random input of a *k*-neuron to originate from a *k*′-neuron, sampled from the adjacency matrices of simulations with *N* = 10^5^. Normalization demands ∑_*k*′_ *f*(*k*, *k*′) = 1. (A) Uncorrelated network, (B) assortative network, (C) disassortative network. The corresponding Pearson coefficients are *p* = −0.004 (uncorrelated); *p* = 0.997 (assortative); *p* = −0.662 (disassortative).

The Pearson degree correlation coefficient is normalized by the variance of the excess in-degree distribution

$${\left(\sigma^{\text{in}}\right)}^{2}=\sum_{k}\;k^{2}Q_{k}^{\text{in}}-{\left(\sum_{k}\;kQ_{k}^{\text{in}}\right)}^{2},$$ (26)

and ranges from -1 for a fully disassortative network up to 1 for a fully assortative network. Note, that Eq (24) suffices to quantify in-degree correlations if in- and out-degrees are equal per neuron. Otherwise, alternative measures may be required [41]. For full simulations, we first construct large random networks with the desired in-degree distribution using a configuration model [46] and then impose degree correlations by shuffling links according to a Metropolis algorithm [47]. For details of the network model, see materials and methods. The three representative networks have *p* = 0.997 (assortative), *p* = −0.004 (uncorrelated) and *p* = −0.662 (disassortative). The connection probabilities *f*(*k*, *k*′) sampled from the adjacency matrices of the three networks are shown in Fig 2.

For the mean-field analysis, it is feasible to use simplified approximations for *f*(*k*, *k*′), so that sampling from full IF networks is not necessary. Additionally, the dimension of the self-consistent Eq (5) may be reduced for efficient computation. For the uncorrelated network, we use Eq (20), which gives

$$f\left(k,k^{\prime }\right)=\frac{Z}{\left\langle k\right\rangle }{k^{\prime }}^{-1}.$$ (27)

In the maximally assortative network, neurons with the same in-degree are connected almost exclusively, and *f*(*k*, *k*′) = *δ*(*k*, *k*′) is a sufficient approximation of the peaks in Fig 2B. The maximally disassortative network segregates into subnetworks where a population of neurons with large in-degree is recurrently connected to a population of neurons with small in-degree. Similar to the assortative network, the probability *f*(*k*, *k*′) of the disassortative network can be approximated by employing a Kronecker delta, using a fit of the peak positions in Fig 2C, which yields $k=\frac{k_{\text{min}}\cdot k_{\text{max}}}{k^{\prime }}$. The probability *f*(*k*, *k*′) is then

$$f\left(k,k^{\prime }\right)=\delta \left(k,\frac{k_{\text{min}}\cdot k_{\text{max}}}{k^{\prime }}\right).$$ (28)

**Fig 3:**
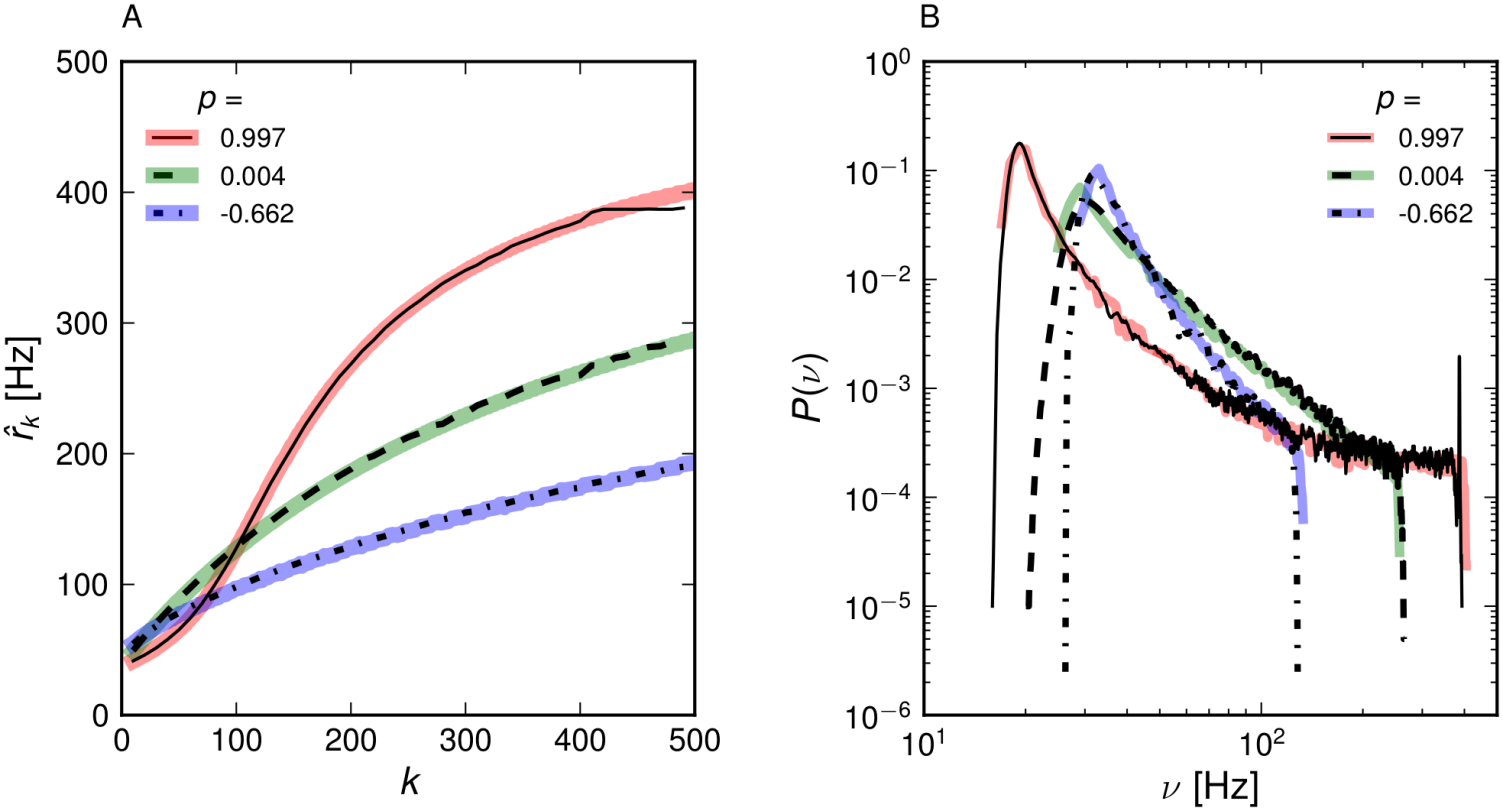
Stationary activity of correlated networks. (A) Population firing rates and (B) distribution of single neuron firing rates for a network with *P*(*k*) ∼ *k* ^−2^, *k* = [10, …, 500] for *s* = 1.2 from simulations (thin full and dotted lines) and theory (thick lines).

**Fig 4:**
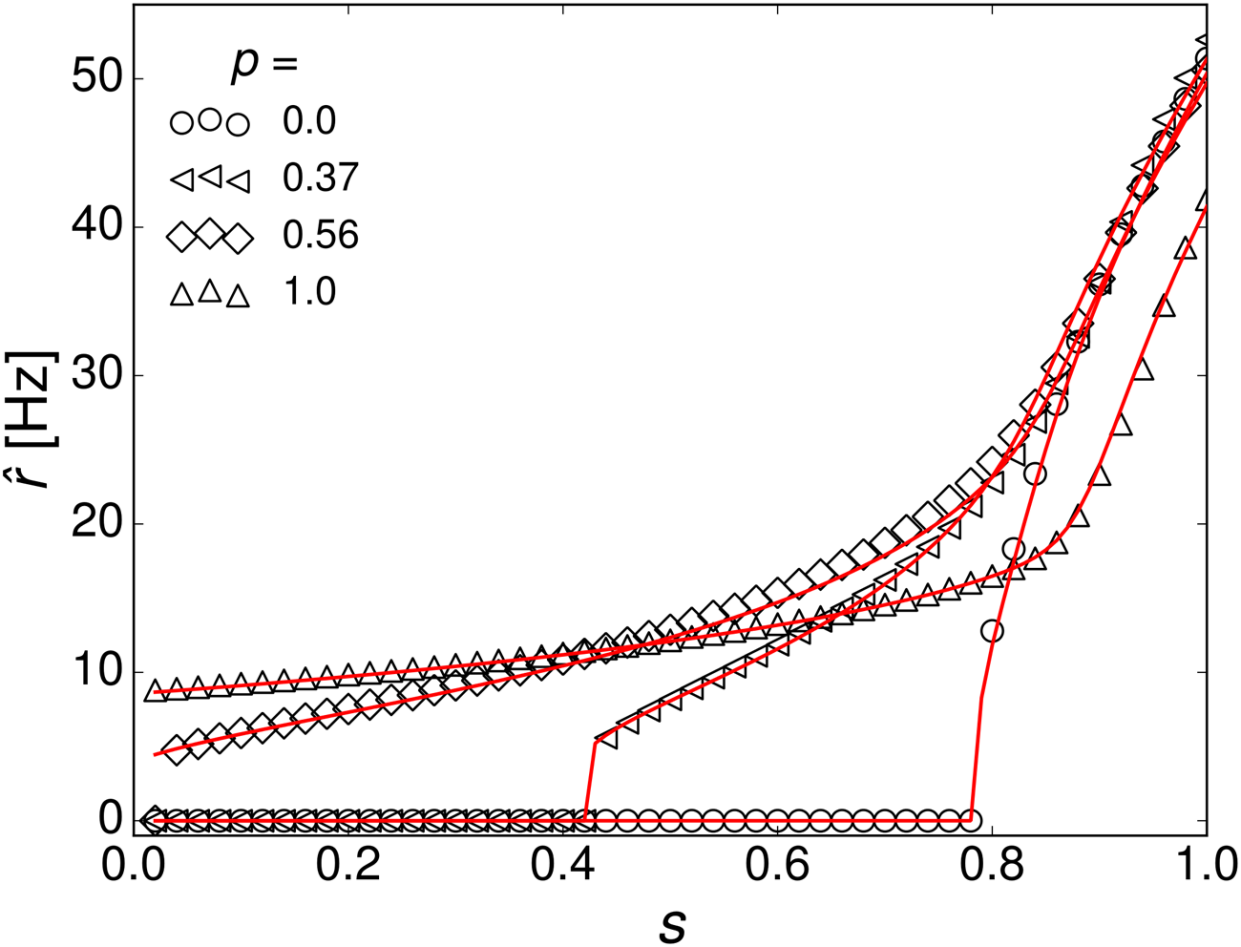
Mean firing rate of the uncorrelated and correlated networks in the sub-threshold regime from simulations (open symbols) and mean-field theory (straight red lines). The joint degree distributions *N*_*kk*′_ for the mean-field calculations were sampled from the adjacency matrices of the constructed networks (*N* = 10^5^).

**Fig 5:**
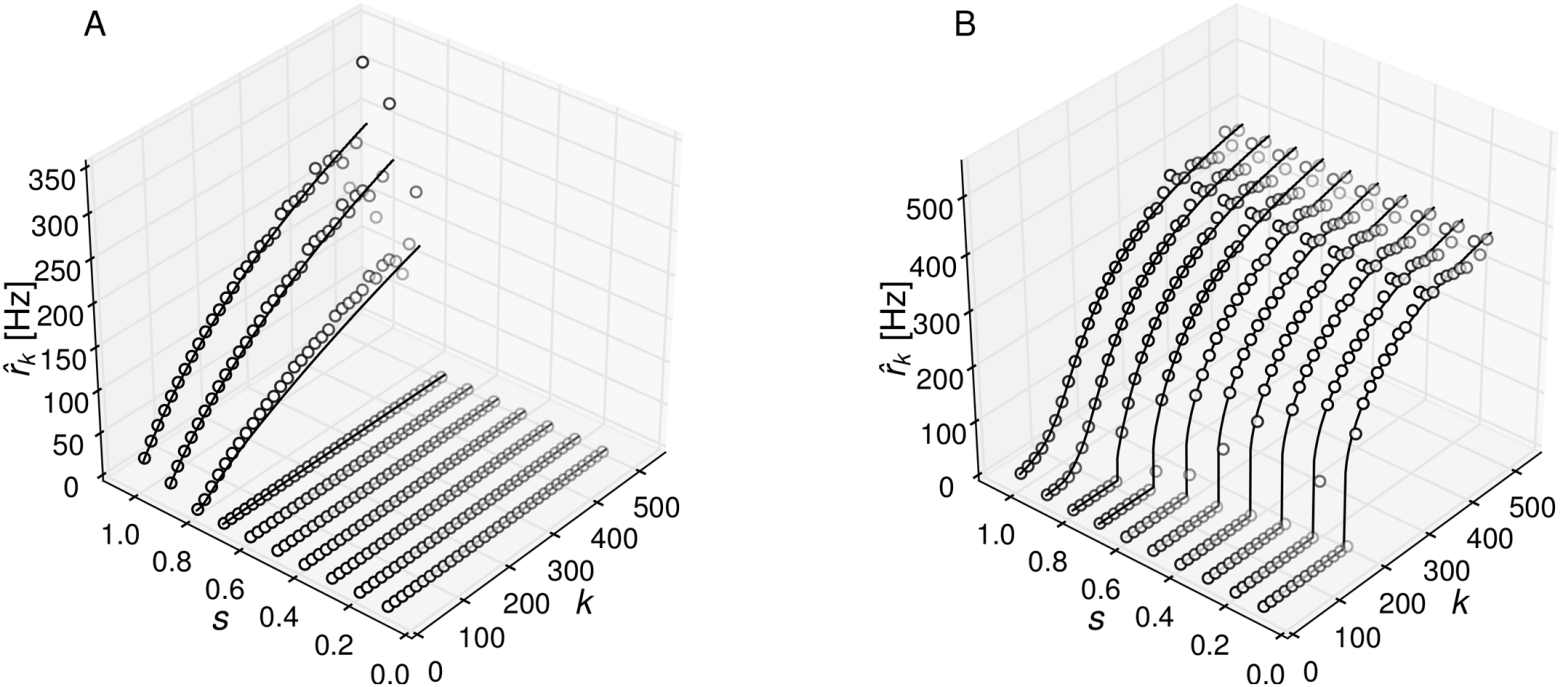
Mean firing rate of the *k*-populations for decreasing sub-threshold stimulus *s*. (A) Uncorrelated network, *p* = 0.000 and (B) strongly assortative network, *p* = 0.996.

Results for the population firing rates ${\hat{r}}_{k}$ corresponding to a stimulus *s* = 1.2 are shown in Fig 3A together with the mean-field predictions. Theory and simulations agree very well. In the assortative network, firing rates of high-degree populations are raised and the ones of low-degree populations are lowered compared to the uncorrelated network. Disassortative networks show the opposite effect. The distribution of single-neuron firing rates *P*(*ν*) can be estimated directly from the population means, if one assumes that the firing rates of all neurons in a given *k*-population are equal. Then, each firing rate ${\hat{r}}_{k}$ occurs *NP* ^*in*^(*k*) times and this distribution of firing rates can simply be binned and normalized. This estimation is close to the actual firing rate distribution in the network (Fig 3B). Assortativity broadens the distribution, whereas disassortativity narrows it. Note, that the firing rate distributions appear to be power-law tailed in disassortative and uncorrelated networks only. Let us now consider the mean firing rate $\hat{r}(s)=\sum_{k}P^{\text{in}}(k){\hat{r}}_{k}$ as a response to a sub-threshold stimulus *s* < 1 for different levels of assortativity. This is plotted in Fig 4 (open symbols) together with theoretical predictions (red lines). In an uncorrelated network $\hat{r}(s)=0$ for small *s* and shows a sharp transition to sustained activity at *s* ≈ 0.8, whereas assortative networks are active even for small *s*. The qualitative explanation is as follows. The neurons with low input degree eventually stop firing when their total input current becomes low. In uncorrelated networks this leads to a cascading failure of spiking of stronger connected neurons. In assortative networks the failure of neurons with low in-degree only leads to failure of the low-degree subnetwork, whereas high-degree subnetworks sustain their recurrent activity (Fig 5). This behavior is reminiscent of findings for percolation in complex networks: At low densities of links, assortative networks remain robust under random failure [48]. It is important to mention that the network may exhibit bistability for very low firing rates, where the mean-field solutions exhibit an additional unstable branch below the stable one that is shown in our results. In practice, bistability leads to hysteresis, where network dynamics depends on previous activity. This effect is discussed extensively in [16, 49]. However, we assume the network to operate in the stable upper branch exclusively by adjusting its sustained activity to changes in the stimulus instead of switching on and off. Networks with strong recurrent activity and the assortative networks considered below will sustain their activity even when the stimulus drops to zero.

### Assortativity Optimizes Information Transfer of Heterogeneous Networks

Our results show that assortativity has a strong impact on the input/output relationship of the model network for small inputs. Here, we are particularly interested in the ability of the network to obtain information about stimuli in this regime and how this ability is affected by assortativity. We quantify the efficacy of this information transfer by the input-output mutual information [50]:

$$I=\int ds\int dr\;P\left(s\right)P\left(r|s\right)\log_{2}(\frac{P(r|s)}{P(r)}).$$ (29)

Here *P*(*s*) is the probability distribution of inputs, which we here assume as an ensemble of stationary stimuli presented to the network, *P*(*r*∣*s*) is the probability distribution of network responses conditioned on the stimulus strength, and *P*(*r*) is the corresponding unconditional probability. The distribution *P*(*r*) could be obtained by collecting a large number of network responses *r*, when the network is subjected to the distribution *P*(*s*) of stimuli over time.

The network response *r* is the average firing rate of *n* randomly chosen neurons, since only a finite fraction of neurons feeds-forward information to a different area of the nervous system for further processing. This response is noisy, because it is sampled from a heterogeneous distribution of single-neuron firing rates. For sufficiently large *n* the variability can be approximated by a Gaussian with mean $\hat{r}$ and variance *σ* ^2^/*n*, where *σ* ^2^ is the variance of mean firing rates in all *k*-populations

$$P\left(r|s\right)=P_{n}\left(r|s\right)=\frac{1}{\sqrt{2\pi \sigma^{2}/n}}\exp(\frac{{\left[r-\hat{r}\left(s\right)\right]}^{2}}{2\sigma^{2}\left(s\right)/n}).$$ (30)

**Fig 6:**
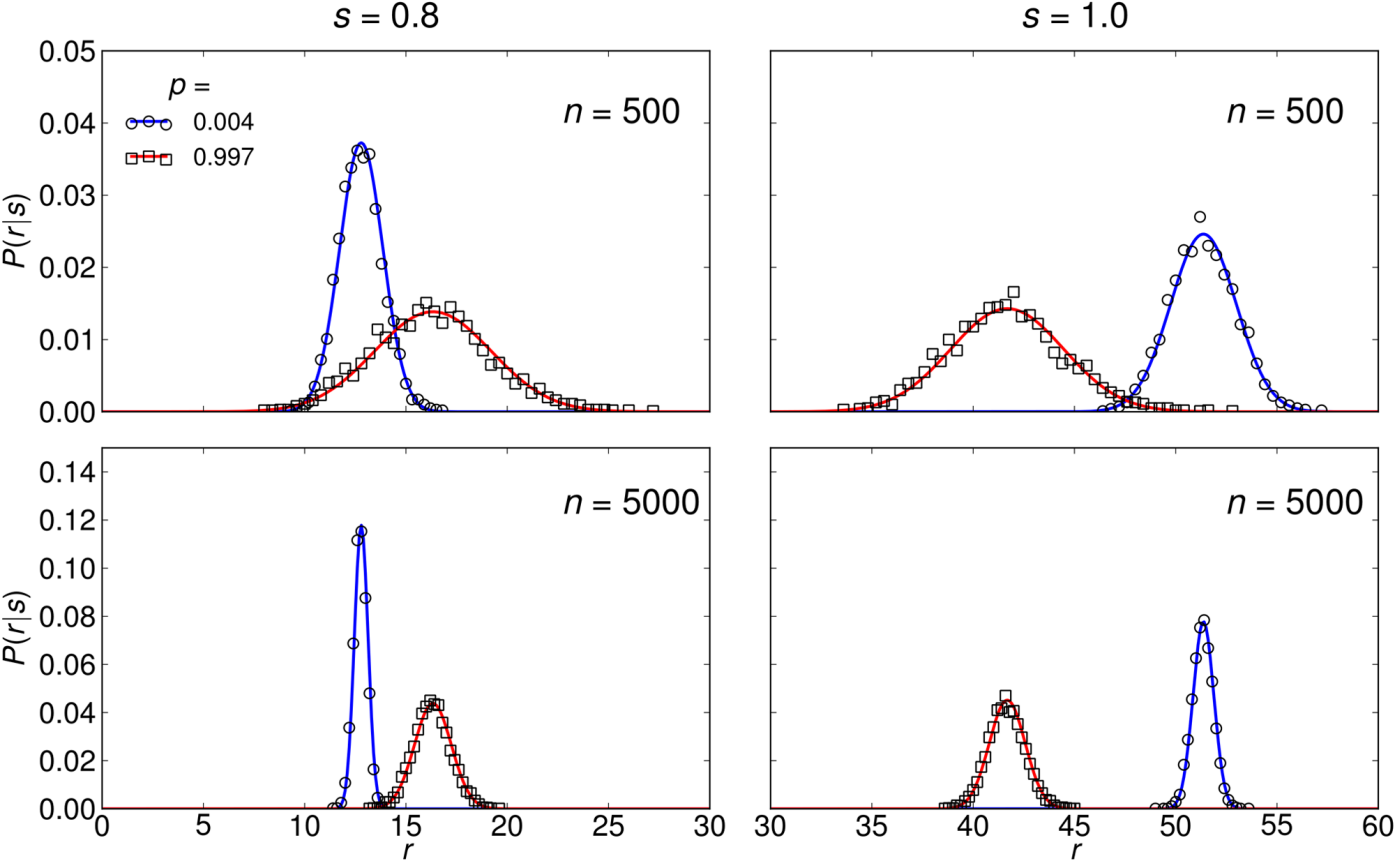
Distribution *P*(*r*∣*s*) of network responses to a stimulus *s*. Normalized histogram of 10^5^ averages of *n* random individual neuron firing rates (symbols), and Eq (30) (lines). For evaluation of Eq (30) we calculated the mean stationary network activity $\hat{r}(s)$ using the self-consistent Eq (5). The corresponding variance of the sampling noise *σ*(*s*)^2^/*n* was obtained from the approximate stationary firing rate distribution as previously discussed for Fig 3B. The distributions are broadened by assortativity, corresponding to larger noise of the response of assortative networks.

Exemplary plots of *P*(*r*∣*s*) from simulations are shown in Fig 6 together with the approximation of Eq (30). Since the distribution of single-neuron firing rates is broadened by assortativity (Fig 3B), the corresponding response *r* is more noisy in the assortative network, arguing that the assortative network is less reliable in encoding the signal. However, assortative networks respond to very weak stimuli (*s* < 0.8), where the uncorrelated network cannot fire, which results in increased sensitivity. Therefore, a quantitative approach is needed to draw conclusion about the signal transmission capabilities of the networks. We calculate *I* using a small noise approximation [51] by expanding Eq (29) as a power series in $\sigma /\sqrt{n}$

$$I=-\int d\hat{r}P\left(\hat{r}\right)\log_{2}\left[P\left(\hat{r}\right)\right]-\frac{1}{2}\int d\hat{r}P\left(\hat{r}\right)\log_{2}\left[2\pi e\sigma^{2}\left(\hat{r}\right)/n\right]+\cdots ,$$ (31)

where the first term (which we denote as *H*) approximates the response variability, or entropy, and the second term corresponds to the noise entropy *H*_noise_ so that *I* = *H* − *H*_noise_. Higher order terms vanish as noise decreases. $P(\hat{r})$ is the probability distribution of mean rates $\hat{r}$ in the absence of sampling noise when the network is exposed to a distribution *P*(*s*) of stimuli

$$P\left(\hat{r}\right)=\int dsP\left(s\right)\delta \left[\hat{r}-\hat{r}\left(s\right)\right]={\left(\frac{d\hat{r}\left(s\right)}{ds}\right)}^{-1}P[s=s\left(\hat{r}\right)].$$ (32)

**Fig 7:**
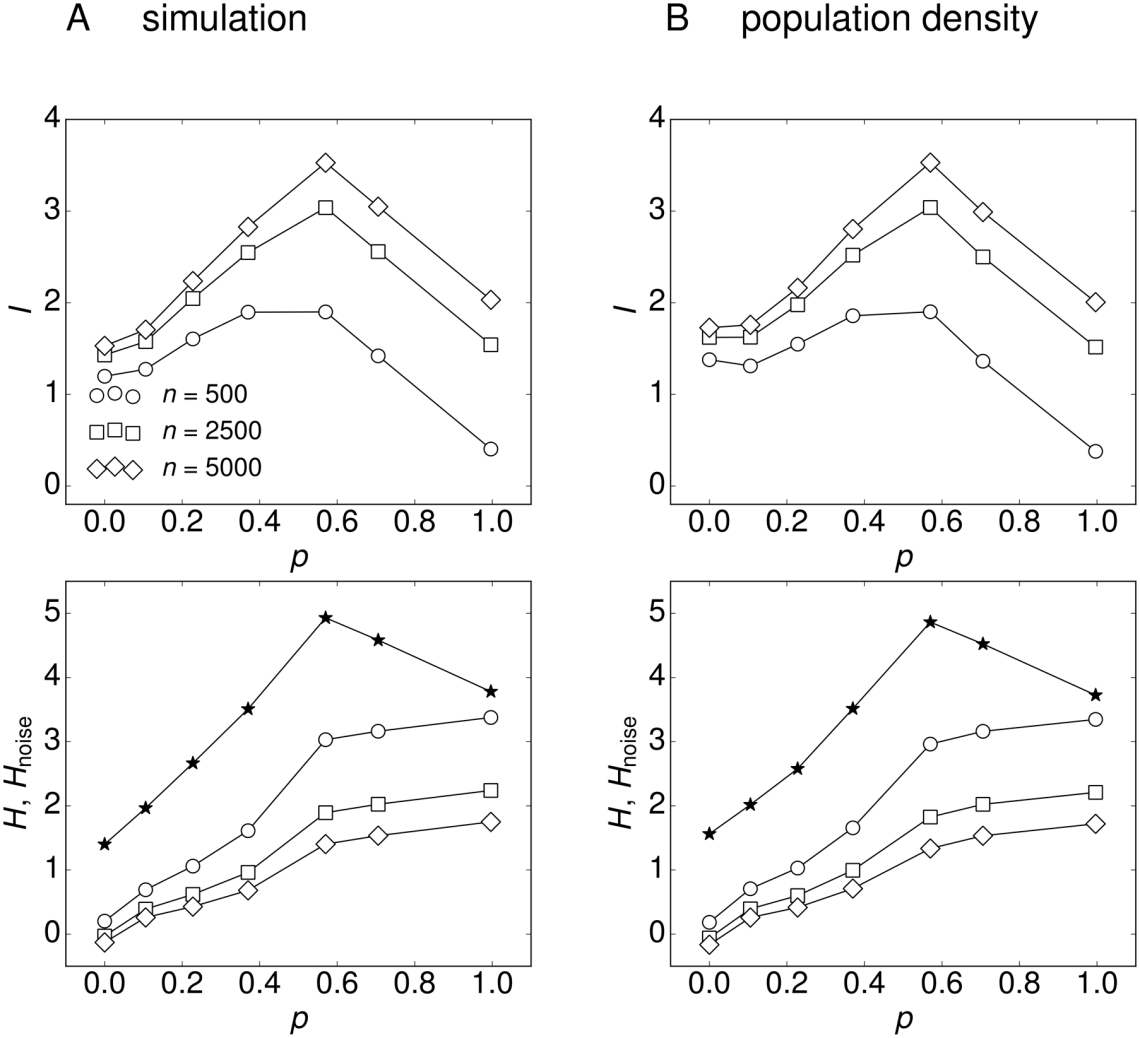
Information transfer of assortative networks. (Top row) Mutual information *I* = *H* − *H*_noise_ of the input/output relation is optimized for intermediate assortativity. (Bottom row) Response variability *H* (stars) and noise entropy *H*_noise_ (open symbols). Network output is quantified by the average firing rate of *n* randomly chosen neurons. (A) Numerical simulations of networks with *N* = 10^5^ neurons and (B) calculations with the population-density approach, where *N*_*kk*′_ is sampled from the adjacency matrices of the correlated networks.

We make the simplifying assumption, that all sub-threshold stimuli are equally likely, *P*(*s*) = const, 0 < *s* < 1. Then, $P(\hat{r})$ follows from the average response function $\hat{r}(s)$, Fig 4, and the associated sampling noise. We found that *I* is optimized for networks with intermediate degree of assortativity, *p* ∼ 0.6 (Fig 7, top row). First, some amount of assortativity increases sensitivity to weak stimuli, which is related to the fact that the response curves in Fig 5 assume a more linear functional dependence. Second, in extremely assortative networks, the network response approaches an almost constant value for different stimuli and thus does not contain much information about the inputs. Additionally, the increase in noise entropy with increasing assortativity reduces *I* even further (Fig 7, bottom row), indicating that the network response is too noisy to reliably transmit information about the stimulus.

In conclusion, the ability of the neuronal network to transmit information is characterized by the shape of the response curve $\hat{r}(s)$ and the variance *σ*(*s*) of the firing rate distribution of the neurons. These features are largely controlled by the degree distribution and degree correlations: Increasing the mean degree of the network results in stronger recurrent activity and a decreased stimulus threshold at which the network begins to fire. In addition, the variance of the degree distribution and degree correlations have a large impact on the shape of the response curve and the firing rate distribution. A larger variance of the degree distribution results in a broader firing rate distribution and increased noise in the signal transmission. Assortative degree correlations further increase the output noise, but also smooth the response curve and increase the sensitivity of the network to low stimuli due to a decreased stimulus threshold. In the following, we investigate the signal transmission capabilities of the networks with respect to changes in their degree distributions and discuss the robustness of our findings.

**Fig 8:**
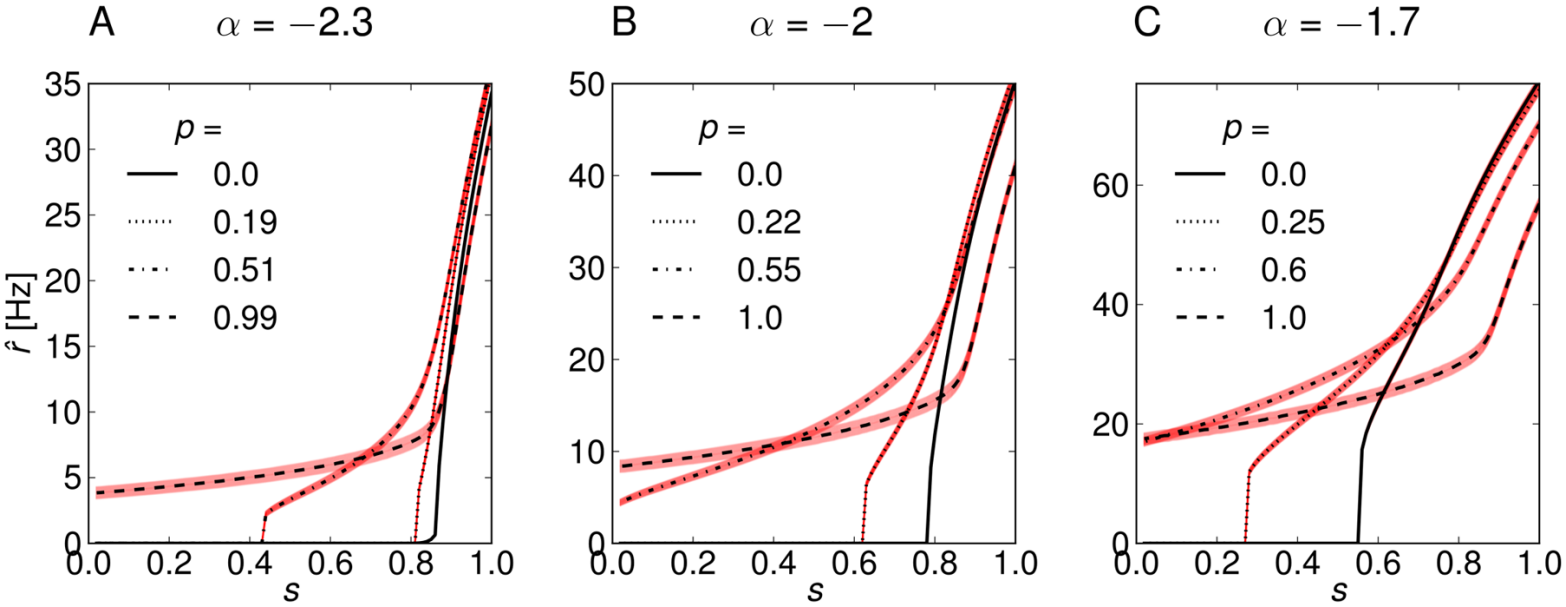
Response curves of three model networks with power-law in-degree distribution *P* ^in^(*k*) ∼ *k* ^*α*^, *k* = [10, … 500] and slightly different exponents *α*. For each network we show the response curves for four different levels of assortativity (thin black lines). The thick light red lines indicate ±1SD of the noise of the output from *n* = 5000 neurons. (A) Model network with large negative exponent of *α* = −2.3 and small mean degree. The network responses to sub-threshold stimuli are very weak due to small recurrent activity. The uncorrelated network (*p* = 0) begins to fire above the stimulus threshold of *s* > 0.8. This threshold is reduced for increasing assortativity, and the response becomes very noisy. (B) Model network with intermediate exponent *α* = −2 and intermediate mean-degree. The network responses are stronger than for *α* = −2.3, but the stimulus threshold for the uncorrelated network is similar at *s* ≃ 0.8. (C) Model network with small negative exponent and large mean degree. The network fires at high rates and has a low stimulus threshold *s* ≃ 0.55 due to strong recurrent activity.

In Fig 8 we show the response curves and the associated standard deviation of the output $\sigma /\sqrt{n}$ for *n* = 5000 of three model networks with power-law distribution between *k*_min_ = 10 and *k*_max_ = 500 and increasing exponents *α* = (−2.3, −2, −1.7). The response curves and variances of the firing rate distributions were calculated with the mean-field approach. The joint degree distributions *N*_*kk*′_ were sampled from the adjacency matrices of the networks. The means ⟨*k*⟩ and variances $\sigma_{P}^{2}$ of the degree distributions are shown in Table 1. The first network with large negative exponent *α* = −2.3 and small mean degree fires at very low rates (Fig 8A), which corresponds to a low response variability. Assortativity changes the shape of the response curve and decreases the stimulus threshold, but strongly increases the output noise. The network with small negative exponent *α* = −1.7 and large mean degree fires at high rates, and the stimulus threshold for the uncorrelated network is shifted to a lower value, *s* ≃ 0.55 (Fig 8C). Hence, this network has a large response variability even when no in-degree correlations are present. For this network, weaker levels of assortativity are sufficient to reshape the response curve and lower the stimulus threshold even further. For all three networks, the noise level is strongly increased by assortativity.

**Table 1:** Mean and variance of the in-degree distribution *P* ^in^(*k*) ∼ *k* ^*α*^, and *k*_min_ = 10 and *k*_max_ = 500.

*α*	⟨*k*⟩	$\sigma_{P}^{2}$
-2.3	29	1707
-2	38	3283
-1.7	54	6001

**Fig 9:**
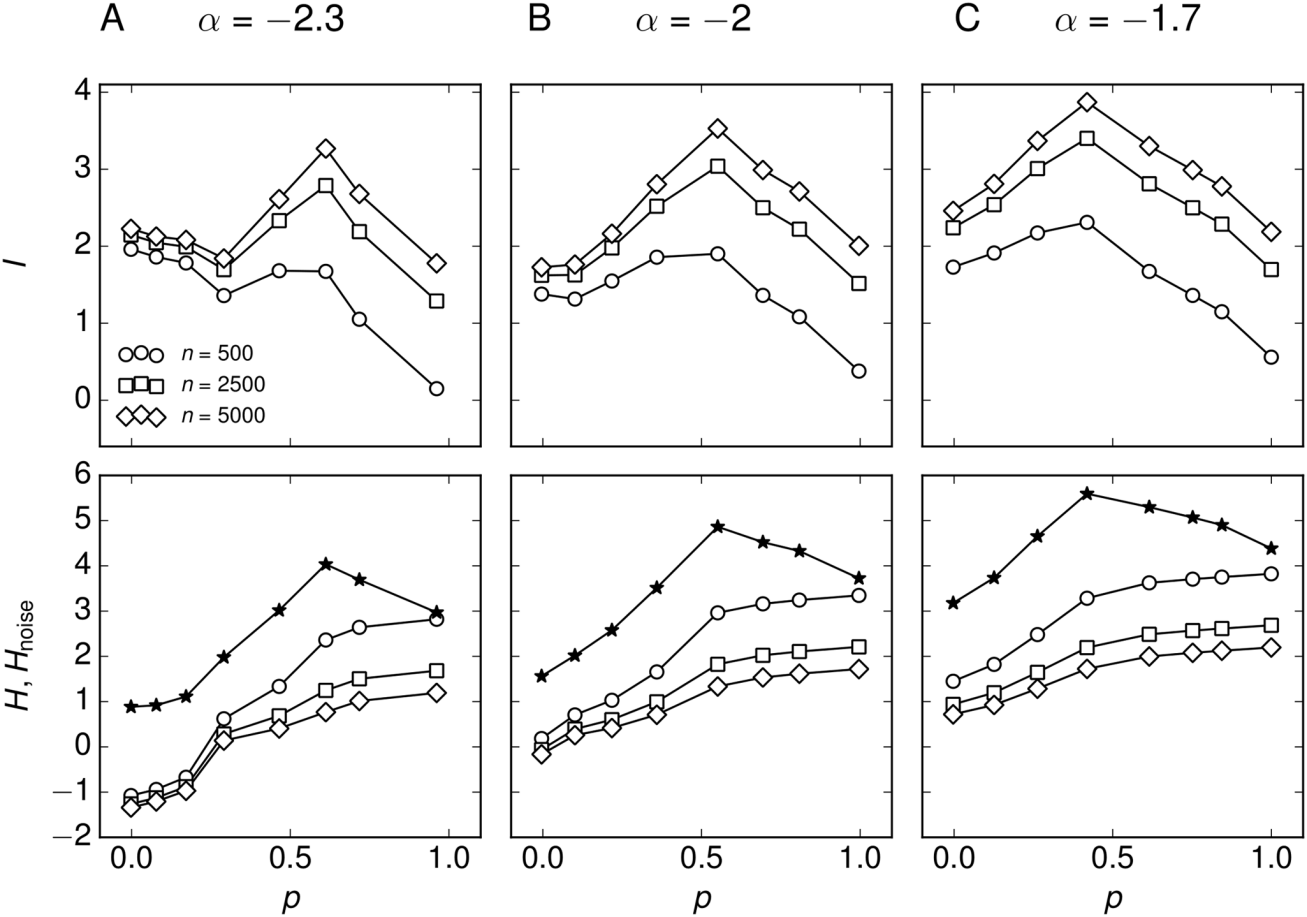
Information transfer of the correlated networks. (Top row) Mutual information *I* = *H* − *H*_noise_ of the input/output relation of the networks. (Bottom row) Entropy *H* (stars) and noise entropy *H*_noise_ (open symbols). (A) The network with large negative exponent *α* = −2.3 has an optimum in information transfer for slightly higher value of *p* compared to the other networks. Additionally, its signal transmission capabilities are poorer which is characterized by lower values of *I* that result from small entropy *H* due to low firing rates. (B) The network with intermediate exponent *α* = −2.3 has its signal transmission optimized at an intermediate value of assortativity, *p* ≃ 0.6. (C) The network with small negative exponent *α* = −1.7 exhibits the most efficient signal transmission. The mutual information peaks for a relatively low value of assortativity *p* ≃ 0.4, which means that the uncorrelated network is already quite efficient in signal transmission.

In the following we compare the three networks with respect to their input-output mutual information (Fig 9). First, the maximum value of mutual information increases slightly for increasing *α* (top row in Fig 9), primarily because increasing *α* increases the mean degree, leading to larger recurrent activity and higher firing rates of the neurons. Increased firing rates correspond to a larger response variability of the networks, e.g. the range of mean responses for *α* = −2.3 (0–35 Hz) is smaller than for *α* = −2 (0–50 Hz), Fig 8A and 8B. However, increasing *α* also increases the variance of the degree distribution, and hence, increases the noise of the network response to a signal. The larger response variability and noise are represented by a larger entropy *H* and noise entropy *H*_noise_, respectively (bottom row in Fig 9). Second, increasing *α* shifts the peak position of the mutual information to lower *p*. This indicates that less assortativity is needed for networks with larger mean-degree and variance of the degree distribution to optimize the information transfer. We conclude that there is a range of network configurations that have their signal transmission optimized by assortative degree correlations, but the optimal level of assortativity depends on the degree distribution of the network. Finally, the optimum vanishes for degree distributions with extremely small or extremely large mean and variance (data not shown). In the former case the mutual information of assortative networks is greatly reduced by exceeding noise levels, because only very few strongly connected neurons sustain firing for small inputs and the vast majority of neurons do not fire. In the latter case, most of the neurons already sustain firing for low inputs in the uncorrelated network, so that assortative degree correlations do not increase the response variability.

## Discussion

Recurrent connectivity is an important property of neuronal circuits, which shapes the dynamics of a complex neuronal network in combination with the external input [29]. Although inter-area connectivity plays a dominant role for cognitive functions, in many parts of the nervous system, particularly the neocortex, excitatory recurrent activity is expected to play a significant role in neuronal computations [29]. One of the most interesting functions of feedback through synaptic links within a circuit is the amplification of small input signals. The general questions we pursue are: (i) How does the sensitivity to low-amplitude signals depend on the network properties and (ii) Can optimization in the topology be achieved for a fixed number of connections in the network? As a first step in this analysis we here presented a generalized mean-field approach to calculate the firing rates of excitatory networks with different topologies of the recurrent connections. Our method is based on dividing the network into populations of neurons with equal in-degrees and solving a system of coupled self-consistent equations for all those populations. In general, the method can be applied to all types of complex neuronal networks that have a limited in- and out-degree and are large and random enough so that the central limit theorem can be applied for each population of neurons.

The structural properties we consider are captured by a single matrix, the joint degree distribution *N*_*kk*′_, which is the average number of connections from a *k*′-neuron to a *k*-neuron. Hence, the impact on network topology on its steady-state dynamics can be easily assessed by examining this distribution. For low external input, the network sustains firing through recurrent activity and the effect of network topology on dynamics becomes very pronounced. In this regime, assortativity increases the sensitivity of a network to very low external inputs, where uncorrelated networks or disconnected neurons would not fire. This effect is similar, but not equivalent, to the smoothing of the single neuron response curve due to increased noise, e.g. from balanced excitatory and inhibitory background activity [52]. Increased noise improves the network sensitivity to small inputs so that less assortativity would be needed to optimize information transfer. In contrast to balanced background activity, purely excitatory background activity would shift the reponse curve to lower values of *s* with a similar effect: less assortativity would be needed to optimize signal transmission. However, the strategy of neuronal networks to amplify low inputs could be based on a combination of sustained recurrent activity and background noise.

We quantified the information transfer between sub-threshold stimuli and network response by their mutual information, assuming that each sub-threshold stimulus is equally likely. In this case, assortativity increases the ability of our model network to transfer sub-threshold signals due to the improved stimulus-response relationship. In a more general sense, degree correlations could be used to tune the stimulus-response relationship to the specific distribution of stimuli. To the aim of enhancing sub-threshold sensitivity of the network through sustained firing, a much simpler strategy of the brain would be to raise recurrent activity by increasing the mean connectivity of the network. However, tuning the network dynamics by degree correlations has two major advantages: First, it does not requires additional axons and synapses, which optimizes wiring economy and second, the mean firing rate of the network is lower, which decreases energy consumption [53].

Our finding that assortativity increases signal transmission is consistent with recent studies, where assortativity has been found to enhance neural network memory in noisy conditions [54] and increases the information content of heterogeneous directed networks [41]. In contrast, assortativity is known to decrease synchronizability and robustness of complex networks [35, 55, 56]. This contradiction can be explained by the different conditions applied to the networks [54]: Assortativity tends to enhance network performance in bad conditions (low density of links and high noise), whereas disassortative networks perform best in good conditions (high density of links and low noise). In our model ‘bad conditions’ are imposed by low level of external inputs. For strongly heterogeneous networks, an excess of assortativity leads to a decrease of mutual information, partly because of an increase in noise in the firing rate. In this case, there exists an optimum in assortativity with respect to information transfer, which is consistent with recent estimates from neuronal cultures [37, 38].

In another related study, Vasquez, Heouweling and Tiesinga [12] investigated the sensitivity and stability of networks with correlations between in- and out-degrees per neuron. They find that disassortative in-out-degree correlations improve stability of the networks and have no impact on the network sensitivity. While the sensitivity of their network model also relies on amplification of the signal by recurrent activity, they use background noise that drives neuronal activity and is independent of the stimulus. Hence, in their model the network does not operate in the sub-threshold regime, where neurons would not spike without recurrent activity. Contrary to them, the stimulus in our model is the only source of external driving force to the network, so that sustained recurrent activity is important to activate the neurons. Moreover, they investigated correlations between in- and out-degrees of the neurons and assume no correlations between in-degrees of connected neurons, whereas in our study in- and out-degrees are positively correlated and correlations between in-degrees of connected neurons are considered. Their finding that various in-out-degree correlations have no impact on the sensitivity of the networks are thus no contradiction to our results and may even generalize them.

We examined highly simplified model networks so that we could focus on their connectivity structure. Possibly the most relevant feature we neglected in our study is inhibitory connectivity. Our model could easily be extended to include inhibitory neurons by treating them as separate populations. However, one would have to decide weather to focus on the structural properties of the excitatory sub-network [13], or to include the inhibitory neurons as a random subset of neurons in the network, without changing the statistics of the connections [15]. Examining the population dynamics of inhibitory and excitatory neurons goes beyond the scope of this paper, but the dynamics of mixed networks with degree correlations could be approached in further studies. So far, we can only speculate how our results would be affected by including inhibitory neurons in the model: The most important effect leading to an increased signal transmission of correlated networks is their improved ability to sustain firing for low inputs. In a mixed excitatory-inhibitory network (EI), assortativity in the excitatory sub-network (E) would possibly create a similar effect, because recurrent excitatory activity is increased for highly connected neurons, while inhibitory activity remains very low. In a recent study of Roxin [13], the analysis of a rate model revealed that a broad in-degree distribution of E-to-E connections in an EI network promotes oscillations because of higher firing rates of strongly connected neurons: These neurons inject large excitatory currents into the network and raise the mean recurrent activity of the whole excitatory population. Hence, we could expect that assortativity further increases the mean firing rate of the excitatory population in these networks. Another study by Pernice, Deger, Cardanobile and Rotter [15] showed that assortativity increases the firing rate of a network, where a random fraction of the neurons are inhibitory.

On a final note, while our results show that intermediate assortative connectivity correlations optimize signal transmission, it is important to stress that the neuronal activity itself is assumed to be uncorrelated. Research focusing on correlations of activity in uncorrelated networks also shows that moderate levels of correlation can be linked to advantages in information processing [36, 57, 58]. On the one hand, highly correlated activity occurs in networks with strong recurrent excitation and is associated with decreased information transfer [59]. On the other hand, uncorrelated activity in networks with weak recurrent excitation is insufficiently small to carry significant information. Consequently, optimal information transfer has been found in networks of intermediate excitability which operate at criticality [36, 60, 61]. Our results are consistent with these findings: Activity of uncorrelated networks is too low to contain information about low inputs, whereas activity in highly assortative networks is not sensitive enough to changes in the stimulus for optimal information transmission.

## Materials and Methods

### Fokker-Planck Description of Complex Networks of Leaky IF Neurons

We would like to describe a population of leaky integrate-and-fire (LIF) neurons that form a network with assortative (or disassortative) property. First, we need to characterize the network statistically with the aim to extract appropriate means and variances of connectivity. Generally we will classify the neurons according to their in-degree, which will be denoted by *k*. Thus, the network consists of neural subpopulations distinguished by *k*. Now, we count the number of directed links *E*_*kk*′_ that originate from neurons with in-degree *k*′ (*k*′-neurons) and go into neurons with in-degree *k* (*k*-neurons). We are interested in the mean number of *k*′-neurons that synapse into a random *k*-neuron

$$N_{kk^{\prime }}=\frac{kE_{kk^{\prime }}}{\sum_{k^{\prime \prime }}E_{kk^{\prime \prime }}}.$$ (33)

We will refer to *N*_*kk*′_ as the *joint degree distribution*.

#### *k*-Population Density Approach

In the following we want to express the dynamics of a network of integrate and fire neurons subject to the usual evolution equation of their membrane potential $V_{i}^{\left(k\right)}(t)$. Here the superscript *k* denotes the in-degree of the neuron and *i* loops over the neurons of the *k*-population. Then, the equation for the potential is

$$\tau \frac{dV_{i}^{\left(k\right)}\left(t\right)}{dt}=-V_{i}^{\left(k\right)}\left(t\right)+RI_{i}^{\left(k\right)}\left(t\right)$$ (34)

where *τ* = *RC* is a time constant related to an *RC*-circuit. When the voltage reaches a threshold *θ* we assume that a *δ*-function spike is emitted by the neuron and the voltage is reset to a value *V*_reset_ for a constant refractory time *τ*_ref_.

The synaptic input current $I_{i}^{\left(k\right)}(t)$ is given by

$$RI_{i}^{\left(k\right)}\left(t\right)=\tau J\;\sum_{k^{\prime }}\sum_{j}\sum_{l}\;\delta \left(t-t_{j,l}^{\left(k^{\prime }\right)}\right).$$ (35)

Here *J* is the efficacy of synaptic connections, *j* loops over the *k*′-neurons that synapse onto the *k*-neuron *i* (here we assume all synapses to be of equal strength) and $t_{j,l}^{\left(k^{\prime }\right)}$ is the arrival time of the *l*-th spike of the *j*-th *k*′-neuron. Below threshold, the Eq (34) can be integrated analytically:

$$V_{i}^{\left(k\right)}\left(t\right)=J\sum_{k^{\prime }}\sum_{j}\;\sum_{l}\;e^{-(t-t_{j,l}^{\left(k^{\prime }\right)})/\tau }H\left(t-t_{j,l}^{\left(k^{\prime }\right)}\right),$$ (36)

where *H*(⋅) is the Heaviside function. We will now follow the population density approach as described by Deco, Jirsa, Robinson, Breakspear and Friston [31], but, different from them, we treat neurons as distinguishable by their in-degree. Thus, we define the *k*-th probability density by

$$p_{k}\left(v_{k},t\right)dv_{k}=\text{Prob}\;\{V_{i}^{\left(k\right)}\in [v_{k},v_{k}+dv_{k}]\},$$ (37)

which is the probability of a *k*-neuron to have a membrane potential in the interval [*v*_*k*_, *v*_*k*_ + d*v*_*k*_]. We employ a Kramers-Moyal expansion to find the relevant Fokker-Planck equation. For this purpose we need the infinitesimal evolution of the voltage

$$dV_{k}\left(t\right)=J\;\sum_{k^{\prime }}\;N_{kk^{\prime }}{\hat{r}}_{k^{\prime }}\left(t\right)dt-\frac{V_{k}\left(t\right)}{\tau }dt.$$ (38)

Here, ${\hat{r}}_{k^{\prime }}(t)$ is the firing rate of an individual neuron averaged for the *k*′-population. From this equation we can determine the first two moments of depolarization

$$\frac{1}{dt}<dV_{k}>=J\;\sum_{k^{\prime }}\;N_{kk^{\prime }}{\hat{r}}_{k^{\prime }}\left(t\right)-\frac{V_{k}}{\tau }.$$ (39)

and

$$\frac{1}{dt}<dV_{k}^{2}>=J^{2}\;\sum_{k^{\prime }}\;N_{kk^{\prime }}{\hat{r}}_{k^{\prime }}\left(t\right)$$ (40)

From this we find the Fokker-Planck equations

$$\begin{array}{cc} \frac{\partial p_{k}\left(v_{k},t\right)}{\partial t} & =\frac{1}{2\tau }\sigma_{k}^{2}\left(t\right)\frac{\partial^{2}p_{k}\left(v_{k},t\right)}{\partial v_{k}^{2}}\\ & +\frac{\partial }{\partial v_{k}}\left[(\frac{v_{k}-\mu_{k}\left(t\right)}{\tau })\;p_{k}\left(v_{k},t\right)\right], \end{array}$$ (41)

where drift and diffusion coefficients are $\left(-\frac{v_{k}-\mu_{k}(t)}{\tau }\right)$ and $\left(\frac{\sigma_{k}^{2}(t)}{\tau }\right)$ with the input mean and variance

$$\mu_{k}\left(t\right)=J\;\sum_{k^{\prime }}\;N_{kk^{\prime }}{\hat{r}}_{k^{\prime }}\left(t\right)\tau$$ (42)

and

$$\sigma_{k}^{2}\left(t\right)=J^{2}\;\sum_{k^{\prime }}\;N_{kk^{\prime }}{\hat{r}}_{k^{\prime }}\left(t\right)\tau ,$$ (43)

respectively. We should note that the derivation of the system of Fokker-Planck Eq (41) is based on the assumption that each population can be described by a separate probability distribution. In general, the system of coupled Langevin Eq (4) is equivalent to a multivariate Fokker-Planck equation (see [62]). The coupled system of one-dimensional Fokker-Planck Eq (41) can be derived from the multivariate Fokker-Planck equation by separation of variables.

#### Self-Consistent Equations for the Stationary Problem

The Fokker-Planck equations can be written in a conservation-of-probability form:

$$\frac{\partial p_{k}\left(v_{k},t\right)}{\partial t}=-\frac{\partial J_{k}\left(v_{k},t\right)}{\partial v_{k}},$$ (44)

with

$$J_{k}\left(v_{k},t\right)=-\frac{v_{k}-\mu_{k}}{\tau }p_{k}\left(v_{k},t\right)-\frac{\sigma_{k}^{2}}{2\tau }\frac{\partial p_{k}\left(v_{k},t\right)}{\partial v_{k}}.$$ (45)

At the voltage threshold, the stationary solution should vanish,

$$p_{k}\left(\Theta ,t\right)=0$$ (46)

and the probability current *J*_*k*_ should represent the mean firing rate, ${\hat{r}}_{k}$, of the population:

$$\frac{\partial p_{k}\left(\Theta ,t\right)}{\partial V_{k}}=-\frac{2{\hat{r}}_{k}\tau }{\sigma_{k}^{2}}.$$ (47)

At *v*_*k*_ → −∞ we need for the integrability of *p*_*k*_

$$\lim_{v_{k}\to -\infty }p_{k}\left(v_{k},t\right)=\lim_{v_{k}\to -\infty }v_{k}p_{k}\left(v_{k},t\right)=0.$$ (48)

Finally, we need to account for the neurons leaving the threshold at time *t* to be reinjected at the reset potential *V*_r_ after a refractory time *τ*_ref_:

$$\frac{\partial p_{k}\left(v_{k},t\right)}{\partial t}=-\frac{\partial }{\partial v_{k}}\;[J_{k}\left(v_{k},t\right)+{\hat{r}}_{k}\left(t-\tau_{\text{ref}}\right)H\left(v_{k}-V_{r}\right)].$$ (49)

The stationary solution of Eqs (46–49) is

$$\begin{array}{cc} & p_{k}^{\left(s\right)}\left(v_{k}\right)=\frac{2{\hat{r}}_{k}\tau }{\sigma }\exp(-\frac{{\left(v_{k}-\mu_{k}\right)}^{2}}{\sigma_{k}^{2}})\\ & \times \int_{\frac{v_{k}-\mu_{k}}{\sigma_{k}}}^{\frac{\Theta -\mu_{k}}{\sigma_{k}}}H(x-\frac{V_{\text{reset}}-\mu_{k}}{\sigma_{k}})\;e^{x^{2}}dx. \end{array}$$ (50)

The normalization of probability mass requires

$$\int_{-\infty }^{\Theta }p_{k}^{\left(s\right)}\left(v_{k}\right)dv_{k}+{\hat{r}}_{k}\tau_{\text{ref}}=1.$$ (51)

We solve Eqs (50 and 51) for ${\hat{r}}_{k}$ and find

$$\begin{array}{cc} {\hat{r}}_{k} & ={\left[\tau_{\text{ref}}+\tau \sqrt{\pi }\int_{\left(V_{\text{reset}}-\mu_{k}\right)/\sigma_{k}}^{\left(\Theta -\mu_{k}\right)/\sigma_{k}}e^{x^{2}}\left(1+\text{erf}\left(x\right)\right)dx\right]}^{-1}\\ & =\phi_{k}\left({\hat{r}}_{k_{\text{min}}},\cdots ,{\hat{r}}_{k_{\text{max}}},s\right), \end{array}$$ (52)

where $\text{erf}(x)=\frac{2}{\sqrt{\pi }}\int_{0}^{x}e^{-y^{2}}\text{d}x$ is the error function and *k*_min_ ≤ *k* ≤ *k*_max_, with *k*_min_ and *k*_max_ being the minimum and maximum in-degree in the network, respectively.

Note that the functions $\phi_{k}({\hat{r}}_{k_{\text{min}}},\ldots ,{\hat{r}}_{k_{\text{max}}},s)$ depend on the firing rates ${\hat{r}}_{k}$ of all populations and thus couple all firing rates. A self-consistent solution of Eq (52) can be obtained by dynamically solving the coupled system of equations [31]

$$\tau_{x}\frac{d{\hat{r}}_{k}}{dt}=-{\hat{r}}_{k}+\phi_{k}\left({\hat{r}}_{k_{\text{min}}},\cdots ,{\hat{r}}_{k_{\text{max}}},s\right),$$ (53)

where *τ*_*x*_ is a time-constant of appropriate choice (we used *τ*_*x*_ = 3 ms for our simulations) [24, 31].

### Network Model

We apply our *k*-population model to a network with power-law in-degree distribution *P*(*k*) ∼ *k* ^−*γ*^. In real neuronal networks, the power-law dependency is always confined to a region between a minimum and maximum degree *k*_min_ < *k* < *k*_max_, where *k*_max_ cannot exceed the number of neurons of the network. Hence, we use the following degree distribution

$$P\left(k\right)=\{\begin{array}{cc} Z\cdot k^{-\gamma }, & k_{\text{min}}\leq k\leq k_{\text{max}}\\ 0, & \text{else} \end{array},$$ (54)

with the normalization constant

$$Z={\left(\sum_{k=k_{\text{min}}}^{k_{\text{max}}}k^{-\gamma }\right)}^{-1}.$$ (55)

We show exemplary results for the following set of parameters: *γ* = −2, *k*_min_ = 10, *k*_max_ = 500. Simulation parameters resemble typical values found in the cortex: *τ* = 20 ms, *τ*_ref_ = 2 ms, *V*_*r*_ = 10 mV, *J* = 0.1 mV, *N* = 10^5^. The time-step of the integration is 0.01 ms and synaptic delays *D*_*ij*_ are drawn uniformly at random between 0 ms and 6 ms [9]. Random delays are included to prevent synchronized cascading firing of the whole network, which is discussed extensively in [63]. Importantly, they do not alter the steady-state Eq (52). Firing rates of the neurons are obtained by counting their spikes in 1 s simulation.

**Fig 10:**
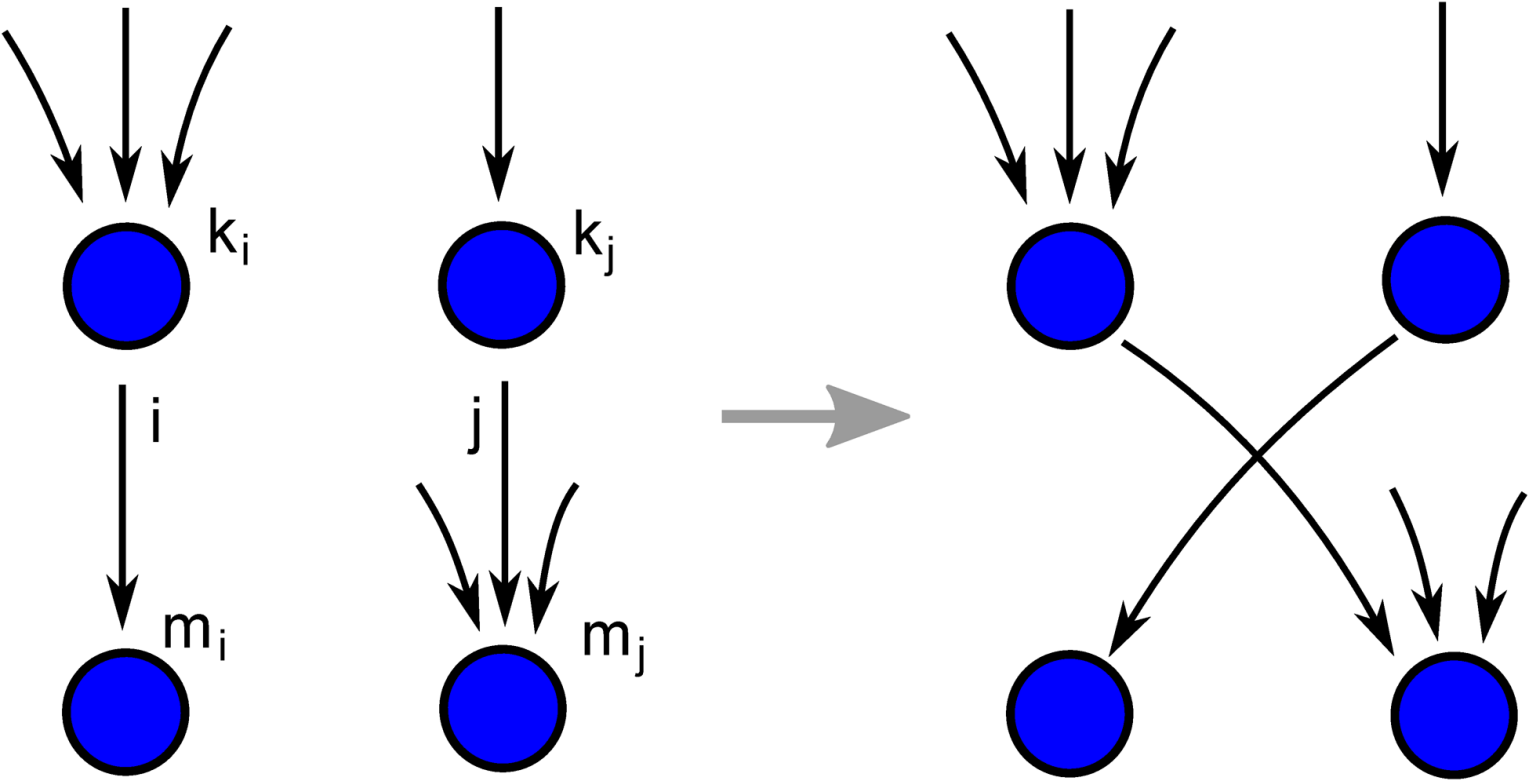
Schematic of a link swap that increases assortative in-degree correlations in the network without changing the in- and out-degrees of the nodes.

For construction of the model network we employ the configuration model of Newman, Strogatz, and Watts [46], which creates random networks with the desired in- and out-degree distribution. In short, the algorithm works as follows: Each neuron of the network is assigned a target in- and out-degree, drawn from the desired degree distribution. The target-degrees of a neuron can be regarded as a number ingoing and outgoing stubs. The algorithm successively connects randomly selected in- and out-stubs until all neurons in the network match their target-degrees with no free stubs left. Self-connections and multiple connections between the same neurons are removed. Then, degree correlations are imposed on the network by a Metropolis algorithm [47], which swaps links according to the in-degrees of the connected neurons. The algorithm randomly selects two links *i*, *j*, originating at neurons with in-degrees *k*_*i*_, *k*_*j*_ and going into neurons with in-degrees *m*_*i*_, *m*_*j*_ (Fig 10). The targets are swapped with probability *g* if the swap increases the desired in-degree correlations of the network. Respectively, the targets are swapped at random with probability 1 − *g*, which reduces existing degree correlations in the network. Thus, the strength of degree correlations can be adjusted by setting a value of *g* between 0 (uncorrelated) and 1 (maximally correlated).

A swap increases assortativity, if

$$k_{i}m_{i}+k_{j}m_{j}<k_{i}m_{j}+k_{j}m_{i}.$$ (56)

A simple schematic for an link swap that increases assortative in-degree correlations is shown in Fig 10. A swap increases disassortativity, if

$$k_{i}m_{i}+k_{j}m_{j}>k_{i}m_{j}+k_{j}m_{i}.$$ (57)

**Fig 11:**
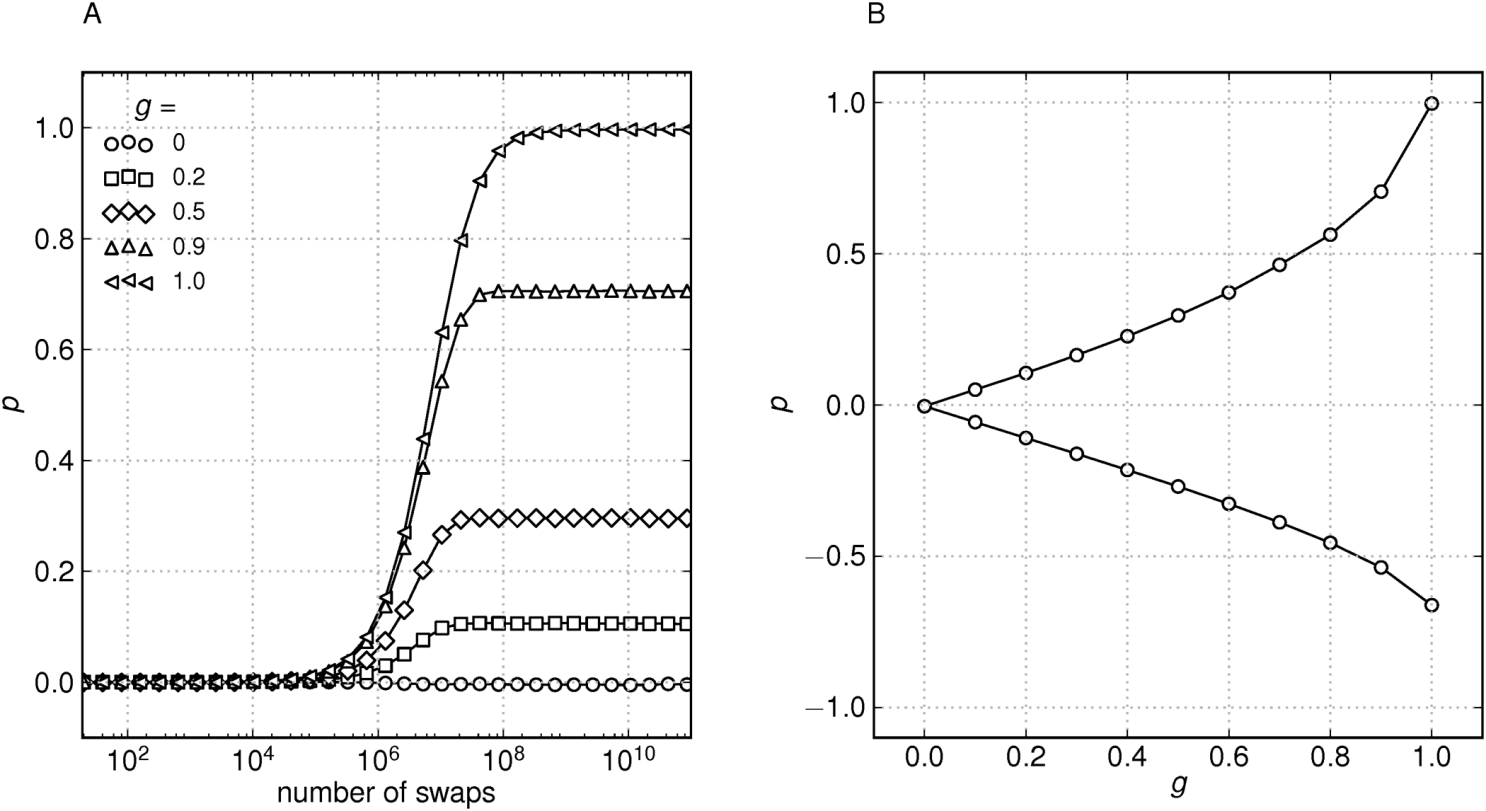
Connectivity correlations of the model network. (A) Pearson degree correlation coefficient for the model network of size *N* = 10^5^ after repeated node swapping for different probabilities *g*. (B) Pearson degree correlation coefficient after saturation (10^10^ swaps).

The swapping procedure is repeated until the network reaches a steady state. We found the steady-state to set in at about 10^9^ iterations for a network of size *N* = 10^5^ (Fig 11).
